# Salivary microRNAs as new molecular markers in cleft lip and palate: a new frontier in molecular medicine

**DOI:** 10.18632/oncotarget.24838

**Published:** 2018-04-10

**Authors:** Vincenzo Grassia, Angela Lombardi, Hiromichi Kawasaki, Carmela Ferri, Letizia Perillo, Laura Mosca, Donatella Delle Cave, Ludovica Nucci, Marina Porcelli, Michele Caraglia

**Affiliations:** ^1^ Mutidiciplinary Department of Medical- Surgical and Dental Specialties, University of Campania “L. Vanvitelli”, Naples, Italy; ^2^ Department of Precision Medicine University of Campania “L. Vanvitelli”, Naples, Italy; ^3^ Drug Discovery Laboratory, Wakunaga Pharmaceutical Co., Ltd., Hiroshima, Japan

**Keywords:** miRNAs, CLP, MTHFR, SATB2, PVRL1

## Abstract

MicroRNAs (miRNAs) are endogenous non-coding RNAs of about twenty-two nucleotides that regulate gene expression through post-transcriptional control. The purpose of the present study was to identify and describe the salivary miRNAs in cleft lip and palate (CLP) patients comparing them with a control healthy group. Twelve patients (mean age 11.9 ± 2.42 years; 6M/6F) formed the study group. The control group was created selecting twelve healthy subjects matched for age and sex with study group. We recorded differences in miRNA expression profile between the saliva of CLP patients and the control group. Specifically, miR-141, miR-223, and miR-324-3p were mostly deregulated between the study and control groups. Interestingly, these three miRNAs are the regulators of the following genes correlated to cleft palate and lip development: *MTHFR*, *SATB2*, *PVRL1*. The present study showed that collecting saliva samples is a non-invasive procedure and is well accepted by CLP patients. MiRNAs can be easily isolated and identified. The differences in regulation of miR-141, miR-223 and miR-324-3p between the two groups of salivary samples suggest that these molecules are valid prognostic biomarkers and therapy dynamic response indicators, also for the accuracy and non-invasive sampling and dosing system.

## INTRODUCTION

Orofacial clefts (cleft lip with or without cleft palate, CL/P, or cleft palate only, CPO) occur with a frequency as high as 1 in 700 live births and are the most prevalent birth defects affecting humans [1, 2]. Patients with CLP have different alterations in facial development associated with functional problems in swallowing, sucking, speaking. The impairment of aesthetics of the face can induce problems in the psychological and social life [3, 4]. Affected infants show an increased risk of death due to associated alterations such as prematurity, respiratory and infection diseases and central nervous system abnormalities. The affected adults have an increased risk of heart disease, suicide, epilepsy and different tumors [5]. Therefore, this malformation syndrome is an important public health problem.

CL/P and CPO are distinct developmental defects with different underlying causes. Genetic and environmental factors contribute to the complex aetiology of CLP. Thirteen loci (gene positions) in different chromosomal regions and 20 genes associated with CLP have been reported from linkage and association analysis. Few studies, however, have addressed the potential involvement of epigenetic factors, such as expression and function of microRNAs (miRNAs) in facial development, and most of these have focused on the developing secondary palate [6–10].

Although multiple molecular mechanisms of epigenetic regulation exist, one of the most actively investigated to date is the action of miRNAs. MiRNAs are short noncoding RNA molecules [11–13] of 19-24 nt that were first identified in 1993 as small RNAs in *Caenorhabditis elegans* [14]. Recent studies indicate that alterations in miRNAs function may play a role in the incidence of these abnormalities [15–17]. MiRNAs regulate expression of genes post-transcriptionally by binding to mRNAs and then inhibiting their translation and/or decreasing their half life [18–20]. The regulation of gene expression by miRNA is performed through the complementation of the miRNA strand with the mRNA target strand, but this occurs in mammals through an imperfect complementarity. This event causes the inhibition of multiple mRNA targets by only one miRNA that, therefore, has potent biological effects in a cell regulating multiple functions. In this light, the expression of different miRNA patterns in a disease can have important effects both in the determination and in the severity of the disease. Therefore, searching for miRNA changes in biological fluids can be important in the diagnosis and prognosis of the disease and in the discovery of new therapeutic targets that can be used to control the disease. In our case, the presence of miRNAs in human saliva has recently become an emerging field for monitoring oral diseases using salivary diagnostics. In fact, saliva is an easily accessible biological fluid that contains miRNAs encapsulated in microvesicles (exosomes) or alternatively bound to proteins such as argonaute 2 (Ago-2) or to lipoproteins such as high density lipoproteins (HDL) [21]. Gallo *et al.* showed in 2012 that in saliva, microRNAs are primarily present inside exosomes rather than associated to other proteins or freely circulating [22]. MiRNAs have been proposed as excellent salivary biomarkers for their easy isolation and identification through conventional quantitative PCR, and as valid prognostic markers and therapy dynamic response indicators, also for the accuracy, non-invasive sampling and dosing systems [23]. Together with transcriptomic and proteomic approaches, miRNAs represent promising diagnostic saliva markers.

In the past few years, several research papers have been published reporting the presence of miRNAs in saliva and their potential as non-invasive biomarkers in oral cancer detection.

Profiling arrays typically require several micrograms or more of input RNA, causing problems with sensitivity and specificity. There are also three different forms of miRNA (pre-miRNAs, pri-miRNAs, and mature miRNAs); thus, the profiling methods have to detect and distinguish between each type.

The identification and characterization of miRNAs in patients with CLP may be useful in obtaining important diagnostic and prognostic information of these craniofacial abnormalities and can be useful to plan the most appropriate therapeutic approach.

In the present study, we have standardized a protocol of sampling and analysis of saliva identifying miRNAs expressed in salivary samples. Moreover, we have compared miRNAs signatures expressed in CLP and healthy patients correlating these miRNAs to genes involved in CLP occurrence.

## RESULTS

**Figure 1:**
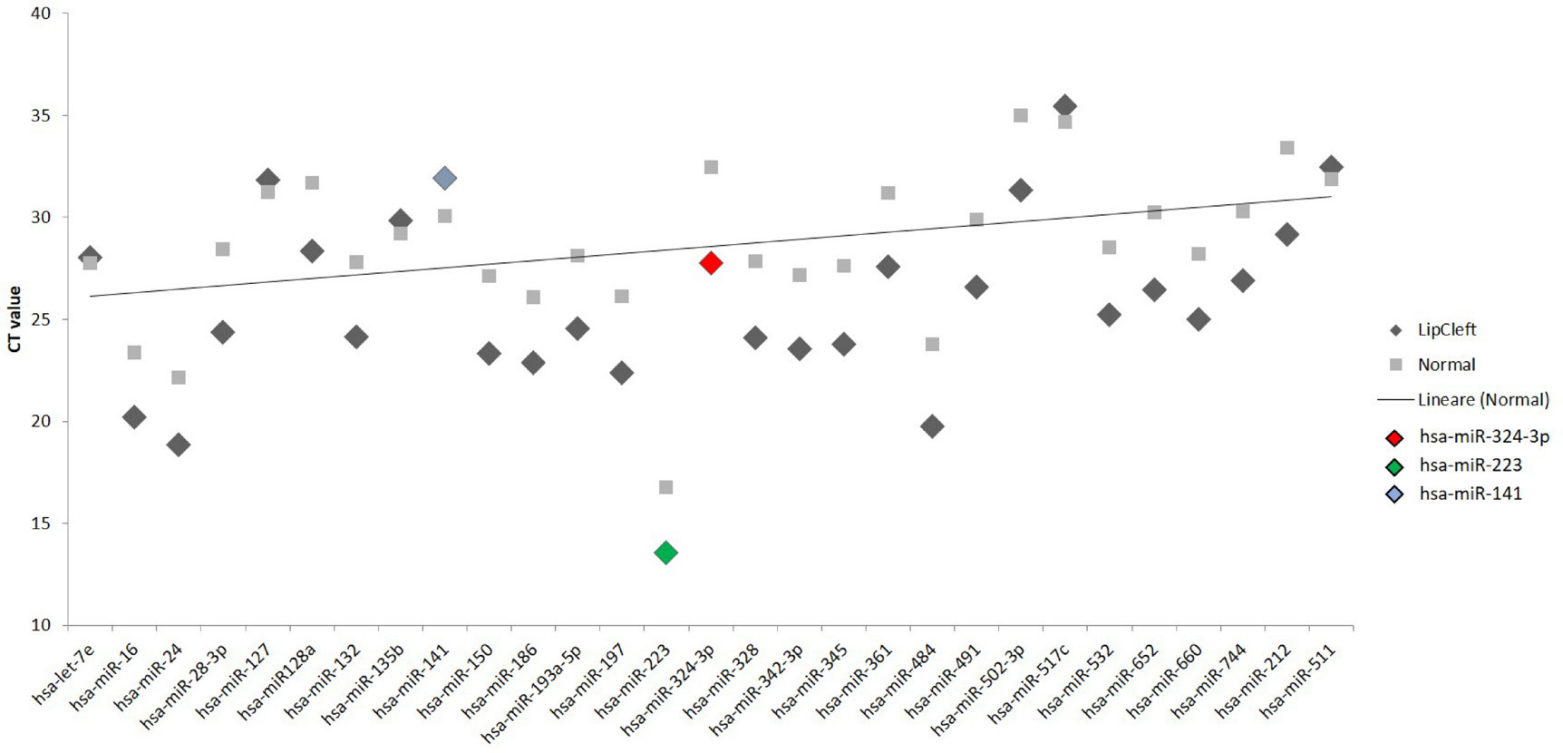
Scatter plot of the expression of the 29 out of 131 different miRNAs found to be deregulated in the patients The fold change was calculated respect to the average ΔCT value of control group. The error bars indicate the mean ± standard deviation (SD).

**Figure 2:**
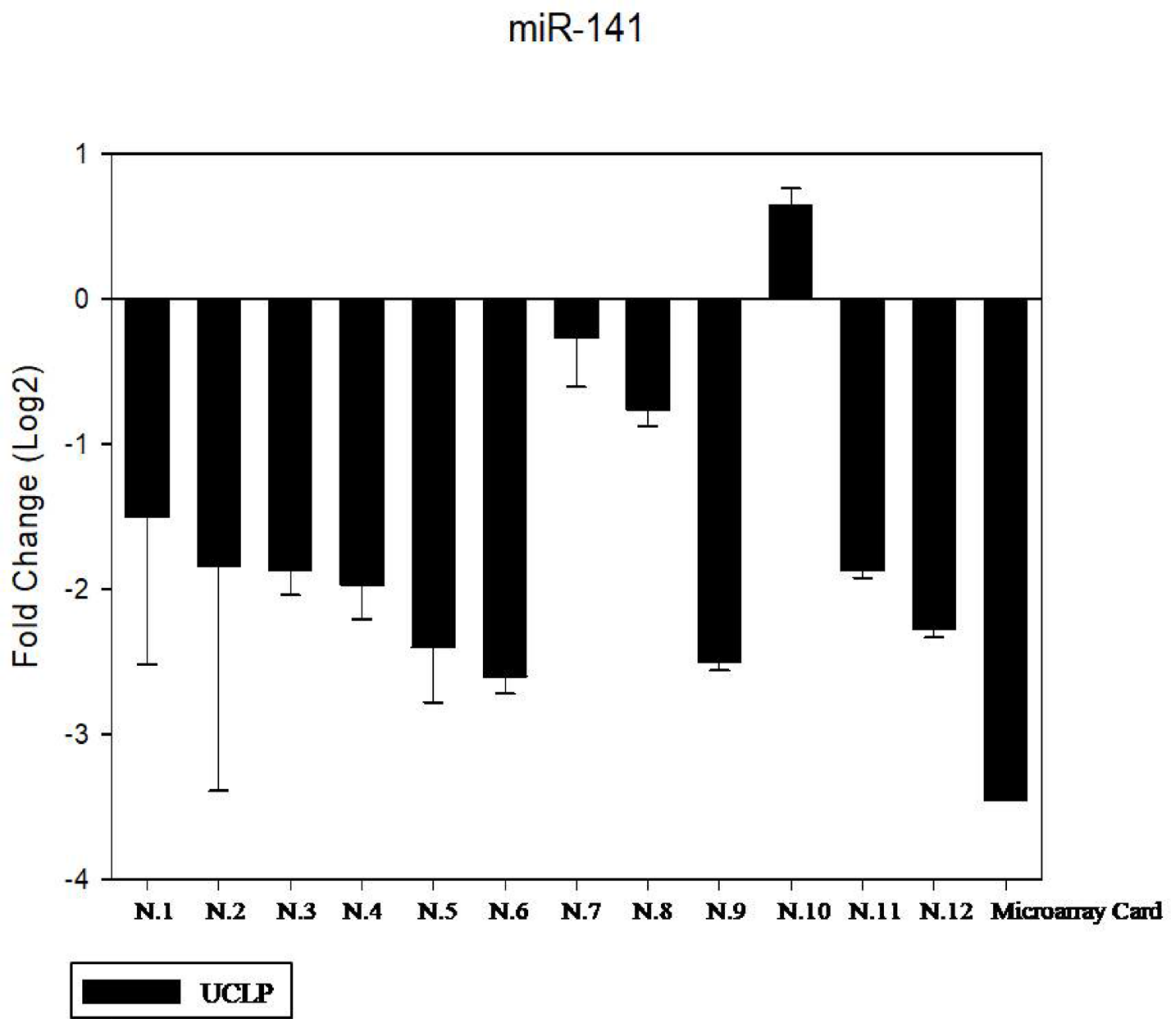
The expression of miR-141 in the saliva of 12 patients with CLP by RT- PCR as compared in the microarray card The fold change was calculated respect to the average ΔCT value of control group. The error bars indicate the mean ± standard deviation (SD).

**Figure 3:**
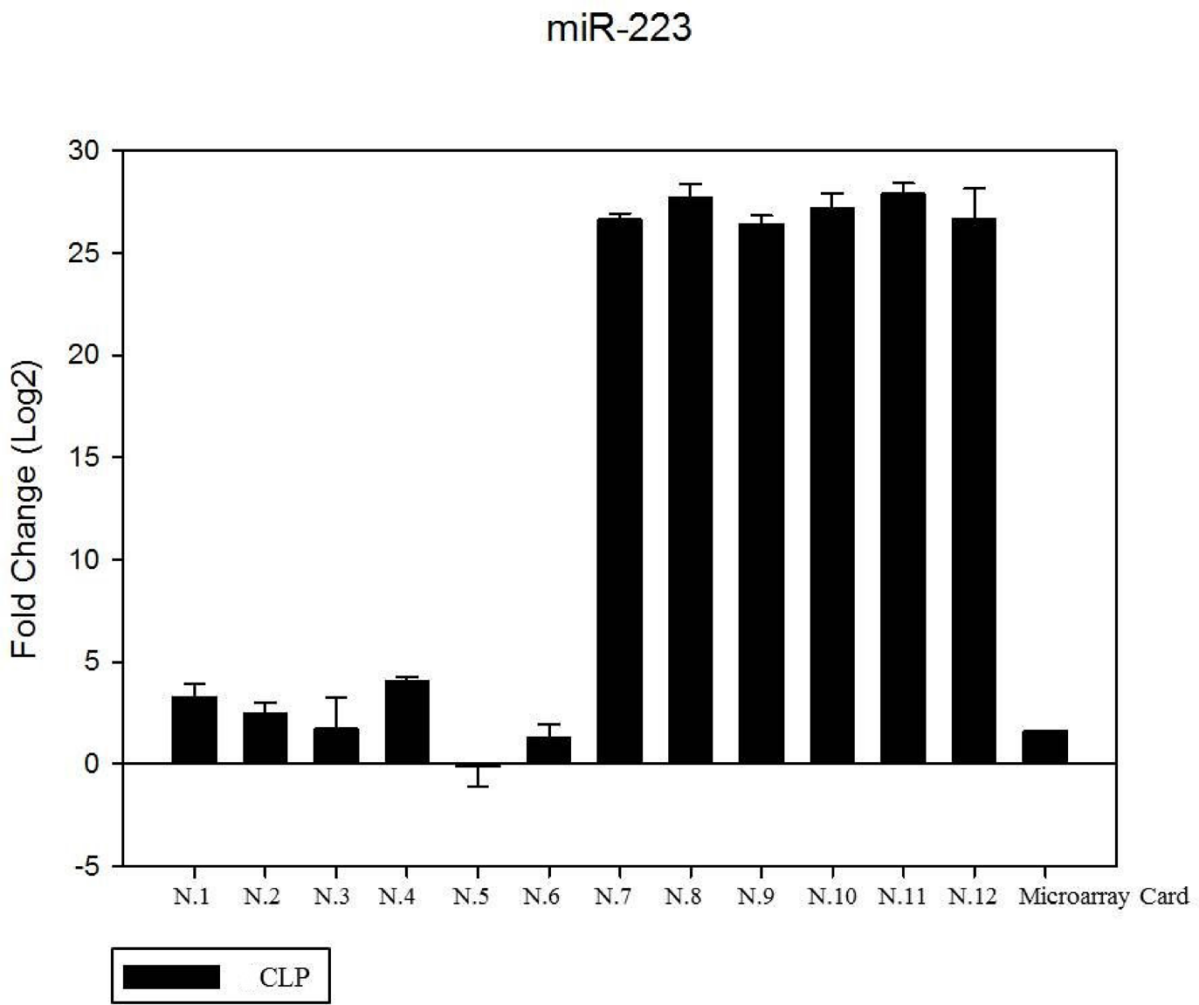
The expression of miR-223 in the saliva of 12 patients with CLP by RT- PCR as compared in the microarray card The fold change was calculated respect to the average ΔCT value of control group. The error bars indicate the mean ± standard deviation (SD).

**Figure 4:**
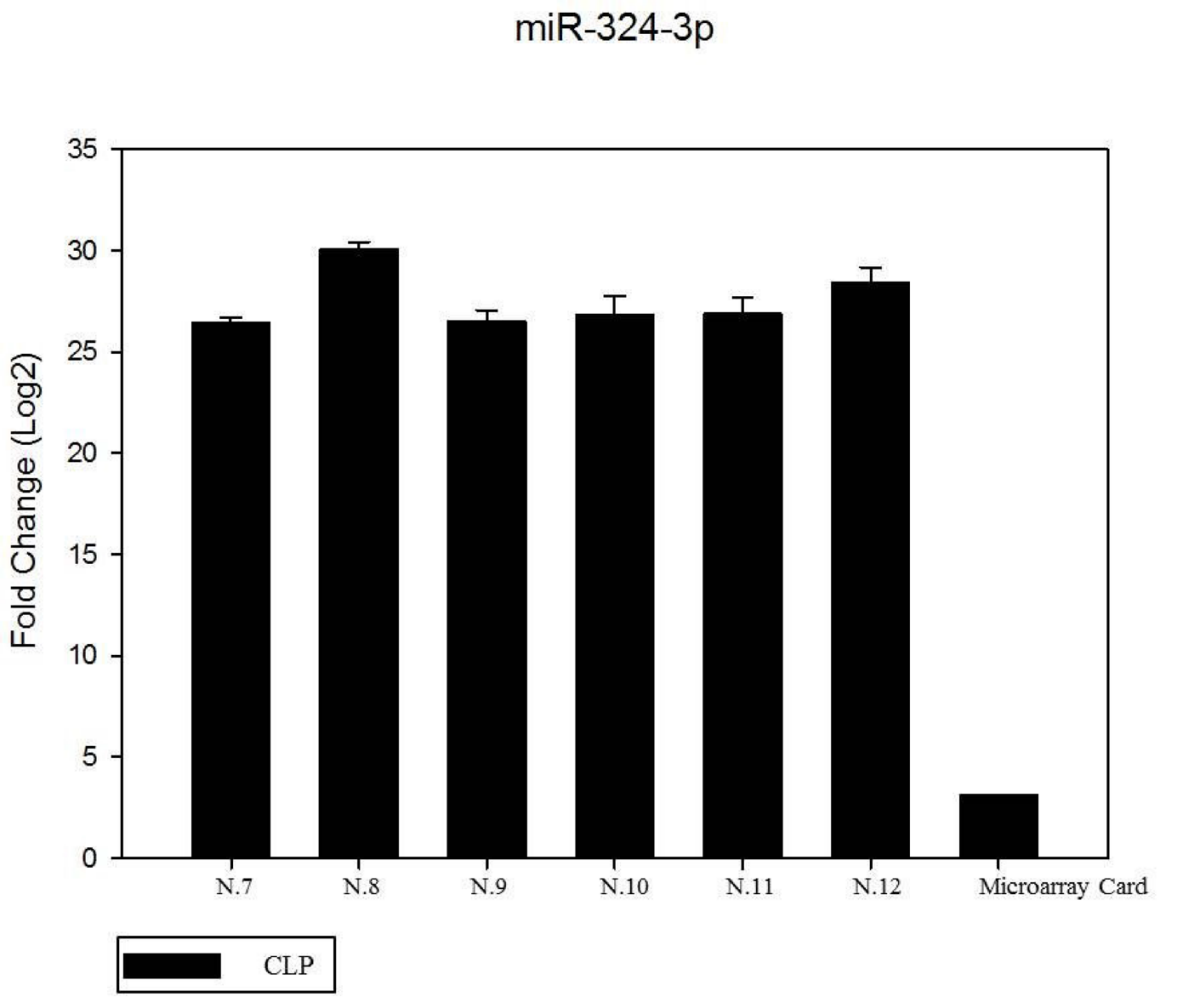
The expression of miR-324-3p in 6 patients by RT- PCR as compared in the microarray card The mean increase of about 27.8 fold; in the remaining 6 patients its up regulation was of about 3.0 fold. (data no shown). The fold change was calculated respect to the average ΔCT value of control group. The error bars indicate the mean ± standard deviation (SD).

**Figure 5:**
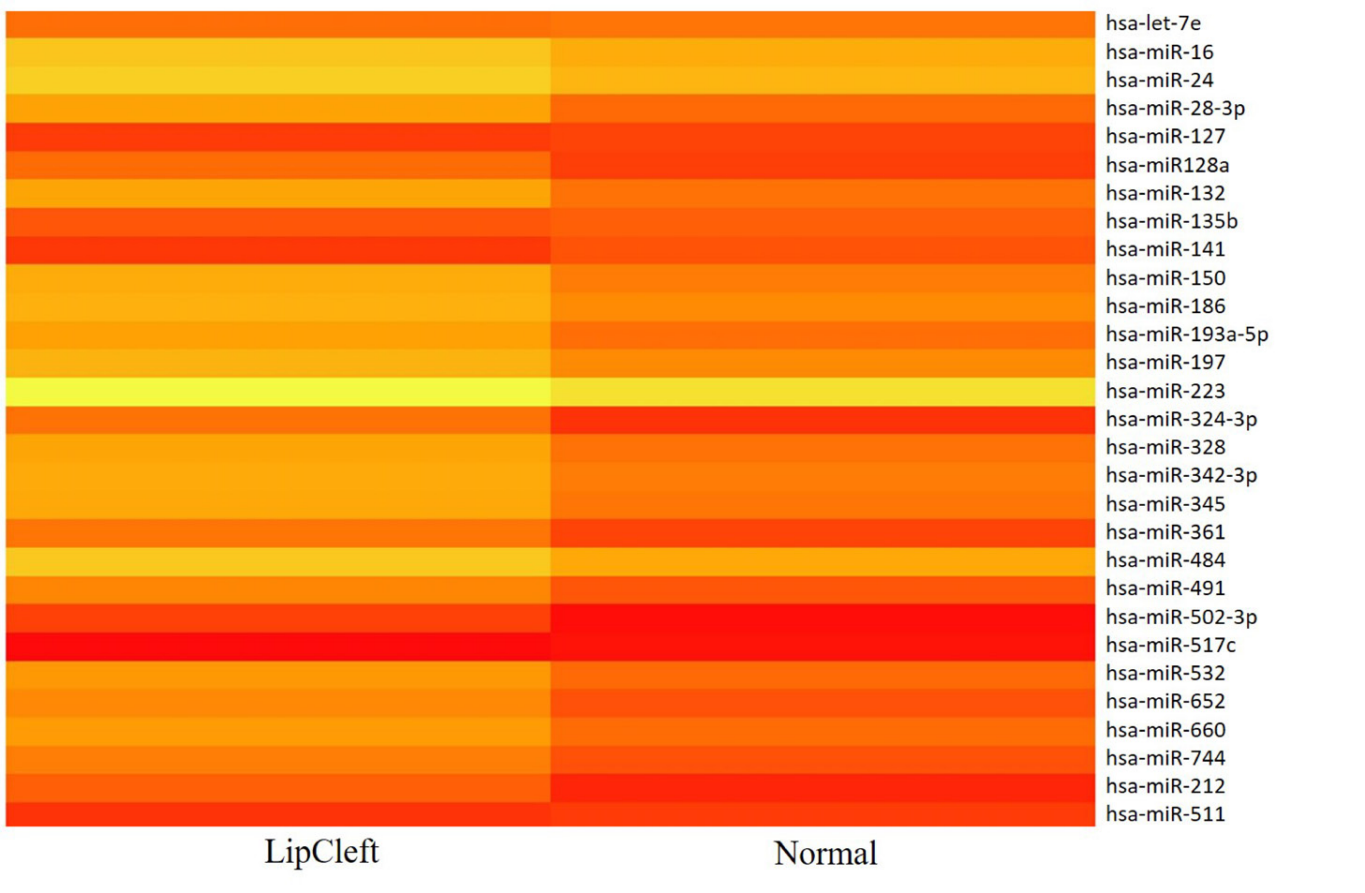
Heat map representation of the 29 out of 131 miRNAs found to be differentially expressed in patients and healthy subjects

We have performed the current study to identify miRNAs expressed in the saliva of patients affected by lip and palatal cleft. We have extracted and purified with the Mirvana PARIS Kit (Thermo Fisher) an amount of 131 miRNAs in the saliva of CLP and control samples. In Table 1 we have reported 29 miRNAs that were significantly differentially expressed between the two groups (more than 1.5-fold change). Three of these miRNAs were selected for the validation with quantitative real time PCR: miR-324-3p and miR-141 (that were the most deregulated) and miR-223 (Figure 1). We selected the most downregulated miRNA (miR-141) with Log2 (2^-(ΔΔCT)^) of –3.45; the most upregulated miRNA (miR-324-3p) with Log2 (2^-(ΔΔCT)^) of 3.12 and a miRNA with not very significantly changed expression (miR-223). We have selected three miR-141 and miR-324-3p miRNAs, which with have high values the of fold changes calculated relatively to the mean value of normal samples and miR-223 as it was previously reported to be involved in embryogenesis [24]. In details, we demonstrated that miR-141 is down regulated in patients with CLP. We analysed 12 patients and we evaluated that in all samples, except on patient number 10, a down regulation of miR-141 was recorded (Figure 2). The decreased expression was statistically significant as compared to the mean expression in healthy controls (*p* < 0.05). Thereafter, we analysed the upregulated miR-324-3p and miR-223. We analysed miR-223 in the saliva of 12 patients and we found that it was upregulated in all samples except in patient number 5 and, therefore, we again validated the results obtained with microarray. (Figure 3) Also in this case, the increased expression was statistically significant as compared to the mean expression in healthy controls (*p* < 0.05). Finally, we analysed miR-324-3p in 6 patients and we have found a significantly up regulation of this miRNA in all samples as compared to microarray analysis. (Figure 4) We observed that miR-223 was up-regulated in all patients but in 6 patients its increased expression was very significant reaching a mean increase of about 27.8 fold; in the remaining 6 patients its upregulation was of about 3.0 fold (Figure 4). For a summary of the different miRNA set expression in the two study populations see also the heat map shown in Figure 5. Moreover, we analyzed the results using the TargetScan program http://www.targetscan.org/, http://pictar.mdc-berlin.de/, http://www.microrna.org/microrna/home.do) that contain the miR-binding site(s) in the UTR. TargetScan analysis predicted the binding of miR-324-3p e miR-223 to the 3′-UTR of methylenetetrahydrofolate reductase (MTHFR) (Table 2).

**Table 1:** Most significant miRNAs expressed in the saliva of CLP

	Saliva						
Target	LipCleft		Normal		ΔΔC_T_	2^–(ΔΔCT)^	Log_2_ (2^–(ΔΔCT)^)
	Mean C_T_	Mean ΔC_T_	Mean C_T_	Mean ΔC_T_	Lipcleft	Lipcleft	Lipcleft
hsa-let-7e	28,03	5,24	27,77	3,41	1,83	0,28	–1,83
hsa-miR-16	20,21	–2,58	23,37	–0,99	–1,59	3,01	1,59
hsa-miR-24	18,83	–3,96	22,15	–2,21	–1,75	3,36	1,75
hsa-miR-28-3p	24,35	1,56	28,44	4,07	–2,51	5,70	2,51
hsa-miR-127	31,82	9,03	31,25	6,89	2,15	0,23	–2,15
hsa-miR-128a	28,35	5,56	31,70	7,34	–1,78	3,42	1,78
hsa-miR-132	24,15	1,36	27,82	3,45	–2,09	4,27	2,09
hsa-miR-135b	29,82	7,03	29,22	4,86	2,18	0,22	–2,18
hsa-miR-141	31,94	9,15	30,06	5,69	3,45	0,09	–3,45
hsa-miR-150	23,31	0,52	27,13	2,77	–2,25	4,76	2,25
hsa-miR-186	22,89	0,10	26,07	1,70	–1,60	3,03	1,60
hsa-miR-193a-5p	24,56	1,77	28,14	3,77	–2,01	4,02	2,01
hsa-miR-197	22,39	–0,40	26,14	1,78	–2,18	4,52	2,18
hsa-miR-223	13,55	–9,24	16,76	–7,61	–1,63	3,10	1,63
hsa-miR-324-3p	27,78	4,99	32,48	8,11	–3,12	8,70	3,12
hsa-miR-328	24,10	1,31	27,85	3,48	–2,17	4,50	2,17
hsa-miR-342-3p	23,54	0,75	27,18	2,81	–2,06	4,18	2,06
hsa-miR-345	23,79	1,00	27,63	3,26	–2,27	4,81	2,27
hsa-miR-361	27,58	4,79	31,20	6,84	–2,05	4,14	2,05
hsa-miR-484	19,77	–3,03	23,78	–0,58	–2,44	5,43	2,44
mmu-miR-491	26,57	3,77	29,90	5,53	–1,76	3,38	1,76
hsa-miR-502-3p	31,31	8,52	35,01	10,65	–2,12	4,36	2,12
hsa-miR-517c	35,43	12,64	34,67	10,31	2,33	0,20	–2,33
hsa-miR-532	25,22	2,43	28,53	4,16	–1,74	3,33	1,74
hsa-miR-652	26,44	3,65	30,26	5,90	–2,24	4,73	2,24
hsa-miR-660	25,01	2,21	28,21	3,85	–1,63	3,10	1,63
hsa-miR-744	26,91	4,12	30,29	5,92	–1,80	3,47	1,80
hsa-miR-212	29,15	6,36	33,43	9,07	–2,71	6,52	2,71
hsa-miR-511	32,48	9,68	31,88	7,52	2,17	0,22	–2,17

Differentially expressed miRNAs between patients affected by lip and palatal cleft and healthy individuals.

**Table 2:** Candidate genes involved in the etiology of nonsyndromic cleft lip and palate and correlation with miRNAs

GENE	MiRNA	References
MTHFR	miR-324-3p (up)	*AM J Med Genet*. 1999 Sep 3;86(1):71–4.
SATB2	miR-141 (down)	*Hum Mol Genet.* 2003 Oct 1;12(19):2491–501.
PVRL1	miR-141 (down)	*Nat. Genet.* 2000, 25:427 -430.
MTHFR PVRL1	miR-223 (up)	*Genet Test Mol Biomarkers* 2016 Jun 1; 20(6): 297–303. *DNA Cell Biol.* 2012 Jul; 31(7):1321–1327.

## DISCUSSION AND CONCLUSION

Every year a large number of children in the world are born with structural birth defects. Despite intellectual and technological strides in the biomedical sciences, including sequencing of the human genome and advances in prenatal care/diagnostics, the underlying causes of nearly 70% of all birth defects and developmental disabilities remain unknown. Isolated oral-facial clefts are among the most common birth defects [1, 2]. Failure of proper fusion between the maxillary and nasal processes can determine cleft lip. Although significant advancements in identifying genes important for secondary palate development have been achieved, similar studies, specifically focused on cleft lip, are only few. In our study we enrolled 24 subjects; 12 with CLP forming the study group (CLP group) and 12 healthy subjects forming the control group (CTR group). The CLP consisted of 6 females and 6 males with mean age of 11.9 ± 2.42 years. The subjects in the control group were matched for number, sex and age (Table 3). Cleft lip and palate is a rare cranio-facial malformation. It occurs in about 1.40 per 1,000 births in Caucasians people. In our region, we have 25,000 newborns per year, so expecting about 30 new CLP cases every year. Only half of the families provide to care CLP patients between 9 and 15 years and many of these choose health facilities outside the region of our hospital. Our interest is to increase the sample size and to run a multicenter study based upon the results found in the present paper that represents a pilot study to standardize the research protocol.

**Table 3:** CLP group and control group

		Male	Female	Mean age ± SD (yr/mo)
**CLP group**	12	6	6	11.9 ±2.42
**Control group**	12	6	6	10.8 ±1.22

We calculated the sample size using the following statistical formula: $n_{1}=n_{2}=2\times {\left(\frac{t_{\alpha }+t_{\beta }}{\delta }\times s\right)}^{2}\;\alpha =0.05\;\beta =0.1\;\delta =2.41$ s = 1.75 with the result = 11.66. On this light, we enrolled 12 patients for each group.

Interestingly, sampling saliva from patients has been well accepted by the subjects enrolled in our study. In according to the age of subjects, parent’s authorization has been requested and in all cases they proved willing to collaborate. Sampling saliva was not perceived as invasive or sticky by either parents or patients themselves even if it must be collected in the morning without breakfast and brunch teeth. Indeed, saliva represents a non-invasive biological fluid sampling, well accepted by patients and easy to apply. The determination of genetic and epigenetic causes of palate alterations is still undefined although exploration of the epigenome—the epigenetic variability in cells (epimutations) — holds the hope for precise diagnosis of disease and congenital anomalies such as cleft lip. A small number of studies up to date have investigated the involvement of epigenetic factors in the determination of this kind of diseases. MiRNAs are among the most actively investigated epigenetic factors to date. Their role in embryonic development is well documented and now widely accepted [25, 26] but their role in development of the midface, particularly the upper lip, is virtually unknown and results of scientific inquiry in this area have only recently begun to emerge [27, 28].^.^ Indeed, the importance of miRNAs for craniofacial development in general, is supported by the severe craniofacial malformations seen in mouse embryos with a conditional deletion of *Dicer* in *Wnt-1* expressing neural crest cells [29]. Loss of *Dicer* expression in *Pax2*-expressing cells also leads to craniofacial defects, including cleft palate and midfacial hypoplasia [30]. The clinical characteristics of the patients were similar but important differences in fold-change between two groups of subjects were found suggesting the existence of different (epi)genetic settings. The number and diversity of targeted genes that result in a cleft probably reflects why CLP is one of the most common features seen in human birth defects. Interestingly, the target prediction identified MTHFR as a target of both miR-324-3p and miR-223. It is reported that MTHFR gene function is decreased by two main MTHFR mutations that researchers focus on most often. These mutations are often called “polymorphisms” and affect genes referred to as MTHFR C677T and MTHFR A1298C. This defect leads to methionine deficiency and over accumulation of homocysteine which, in turn, leads to lowered MTHFR enzymatic activity and/or folate levels. As the result of reduced enzymatic activity and elevated plasma homocysteine levels, the patients may exhibit congenital malformations (tetralogy of Fallot, plus cleft lip and palate). It is probable that upregulated miR-324-3p and miR-223 may decrease the expression and enzyme activity of MTHFR, elevating plasma homocysteine levels. Moreover, TargetScan analysis predicted the binding of miR-141 to the 3′-UTR of Special AT-rich sequence-binding protein 2 (SATB2) and poliovirus receptor-related 1 (PVRL1) (Table 2). The SATB2 is the first cell-type-specific transcription factor that specifically binds nuclear matrix attachment regions (MARs) and is involved in transcriptional regulation and chromatin remodelling. It plays an important role in tooth and craniofacial development [31–34]. Previous studies showed that complete functional loss of SATB2 leads to increased apoptosis in the developing jaw and subsequent down-regulation of the expression of genes (Pax9, Alx4 and Msx1) involved in craniofacial development in humans and mice [35]. It is probably that the downregulation of miR-141 may increase the expression of SATB2 and to be cause of uncorrect craniofacial development. Moreover, PVRL1 is a member of the adhesin family of cell surface proteins that possess an immunoglobulin-related structure, and its main function relates to the formation of tight junctions between epithelial cells [36]. In addition, during development, the pair of palatal shelves must be elevated along with the development of the tongue, after which the palatal epithelium appose and fuse together. PVRL1 plays a crucial role in these processes and its increase may plays a role in the regulation of adherens junctions and tight junctions in epithelial cells.

In conclusion, the present study is the first one investigating on the role of salivary miRNA expression in CLP. CLP patients between 9 and 15 years are subjected to many treatments as bone grafting jaw, orthodontics, additional cosmetic corrections including jawbone surgery, speech therapy, pharyngoplasty. Some of these are very long, complex, difficult and expensive associated to different and critical prognostic evaluations. All therapeutic approaches are guided only by the patient’s clinical analysis and diagnosis without evaluating the genetic and epigenetic variability of these subjects. Our future objective is identifying the different ways to respond to therapy and consequently modulate all therapeutic approaches through not only clinical evaluation but with the epigenetic characterization achieved with a simple saliva collection.

Our data suggest miR-141, miR-324-3p and miR-223 as possible salivary biomarkers of the malformation. A complete understanding of the regulation of expression of all genes involved in schisis development will require the integration of miRNA expression profiles. These include the transcriptome, proteome, methylome, and histone modifications. How these diverse regulatory mechanisms lead to the coordinated morphogenetic processes of CLP development is a prerequisite for developing strategies for the treatment or prevention of these craniofacial abnormalities.

## MATERIALS AND METHODS

### Patient enrolment and selection

Appropriate ethical approval was secured by the Health Research Ethics Board of the University of Campania “L. Vanvitelli” (No.1032).

All CLP patients were consecutively enrolled at the Orthodontics Clinic of the University of Campania “L. Vanvitelli” from January 2015 to January 2016. Patients with syndromes including cleft lip and/or palate were excluded from the study. From an initial sample of 25 patients we have selected 17 patients. Five patients refused to participate in the study so the sample became of 12 final patients.

Anamnesis is of all patients was obtained. For all subjects included in the study intra-oral and extra-oral photographs, craniox-rays in latero-lateral and posterior-anterior view and dental casts were requested and examined. Twelve patients without CLP matching for age and sex formed the control group. The same records and anamnesis were required in these subjects. Parents of all children involved in the study signed an informed consent before the beginning of the study.

### Saliva collection and storage

A protocol for collecting the saliva samples was developed according with the most recent literature in evidence. Following the collection, saliva was processed as soon as possible, to avoid cell damage and subsequent miRNA release from exosomes in saliva. For the isolation of salivary exosomes two centrifuge steps were performed.

### miRNA detection

miRNA extraction was performed using MIRVANA PARIS KIT, (Thermofisher Scientific, MA, USA), it was adapted to our purpose for its higher sensitivity.

Subsequently, using the TaqMan^®^ MicroRNA Reverse Transcription Kit (Thermofisher Scientific) and the Megaplex™ RT Primers, (Thermofisher Scientific) single-stranded cDNA was synthesized from total RNA samples. Then, our cDNA targets were preamplified to increase the quantity of desired cDNA for gene expression analysis using 384-well TaqMan^®^ MicroRNA Arrays. The preamplified target cDNA was amplified by DNA polymerase from the TaqMan^®^ Universal PCRMaster Mixusing sequence-specific primers and probes on the TaqMan^®^ MicroRNA Array. About 100 μL of the PCR reaction mix were dispensed into each port of the TaqMan MiRNA Array. Then, the array was loaded and run on an Applied Biosystems Viia7 instrument by using the default thermal-cycling conditions. The presence of the target was detected in real time through cleavage of TaqMan probe by the polymerase 5′-3′ exonuclease activity. To validate the results of the array cards, the expression of miRNAs found to be differentially expressed in the screening phase of the study was independently determined by real-time PCR (Taqman, Life Technologies). cDNAs were synthesized as detailed above and the expression of individual miRNAs was determined using pre-designed probe-primer sets from Life Technologies. The expression of each miRNA tested was normalized by the ΔΔCt method [37]. To perform the real-time PCR we used TaqMan^®^MicroRNA Assays. They are designed to detect and accurately quantify mature microRNAs (miRNAs) using Applied Biosystems real-time PCR instruments (ViiA7 Life Techonologies Waltham, MA USA). The TaqMan MicroRNA Assays use looped-primer RT-PCR, a new real-time quantification method, to accurately detect mature miRNAs. Single-stranded cDNA were synthesized from total RNA samples using the TaqMan^®^ MicroRNA Reverse Transcription Kit.

During the target amplification step, the AmpliTaq^®^ Gold DNA polymerase amplifies target cDNA synthesized from the RNA sample, using sequence-specific primers from the TaqMan Assay Plates.

### Data analysis

Using comparative CT method, we used endogenous controls to normalize the expression levels of target genes by correcting differences in the amount of cDNA loaded into PCR reactions.

To normalize human total RNA samples, an appropriate constitutively expressed endogenous control was selected.

The 2-ΔΔ*CT* method has been extensively used as a relative quantification strategy for quantitative real-time polymerase chain reaction (qPCR) data analysis. This method is a convenient way to calculate relative gene expression levels between different samples in that it directly uses the threshold cycles (*CTs*) generated by the qPCR system for calculation [38]. Relative quantification was performed using the ΔΔCt method using U6 as housekeeping.

A database system using the Statistical Package for Social Sciences (SPSS) version 18 was made. The descriptive statistical analysis was performed on proportions calculations, measures of central tendency and variability for socio-demographic and clinical aspects.

### miRNA target analysis

Genes that contain the miR-binding site(s) in the UTR were obtained using the TargetScan program (http://www.targetscan.org/, http://pictar.mdc-berlin.de/,http://www.microrna.org/microrna/home.do).

## Abbreviations

CLP: cleft lip and palate
CPO: cleft palate only
miRNAs: microRNAs
MTHFR: methylenetetrahydrofolate reductase
SATB2: Special AT-rich sequence-binding protein 2
PVRL1: poliovirus receptor-related 1
MARs: nuclear matrix attachment regions
SPSS: Statistical Package for Social Sciences
